# No evidence for substrate accumulation in Parkinson brains with *GBA* mutations

**DOI:** 10.1002/mds.26278

**Published:** 2015-06-11

**Authors:** Matthew E. Gegg, Lindsay Sweet, Bing H. Wang, Lamya S. Shihabuddin, Sergio Pablo Sardi, Anthony H.V. Schapira

**Affiliations:** ^1^ Department of Clinical NeuroscienceUCL Institute of NeurologyLondonUK; ^2^ Genzyme, a Sanofi CompanyFraminghamMassachusettsUSA

**Keywords:** glucocerebrosidase, lysosomes, Parkinson's disease, sphingolipids

## Abstract

**Background:**

To establish whether Parkinson's disease (PD) brains previously described to have decreased glucocerebrosidase activity exhibit accumulation of the lysosomal enzyme's substrate, glucosylceramide, or other changes in lipid composition.

**Methods:**

Lipidomic analyses and cholesterol measurements were performed on the putamen (n = 5‐7) and cerebellum (n = 7‐14) of controls, Parkinson's disease brains with heterozygote *GBA1* mutations (PD+GBA), or sporadic PD.

**Results:**

Total glucosylceramide levels were unchanged in both PD+GBA and sporadic PD brains when compared with controls. No changes in glucosylsphingosine (deacetylated glucosylceramide), sphingomyelin, gangliosides (GM2, GM3), or total cholesterol were observed in either putamen or cerebellum.

**Conclusions:**

This study did not demonstrate glucocerebrosidase substrate accumulation in PD brains with heterozygote *GBA1* mutations in areas of the brain with low α‐synuclein pathology. © 2015 The Authors. Movement Disorders published by Wiley Periodicals, Inc. on behalf of International Parkinson and Movement Disorder Society.

Mutations in the *GBA1* gene are numerically the most important genetic risk factor for developing Parkinson's disease (PD).^1^, ^2^ A large multicenter study showed that PD patients had an odds ratio of over 5 for carrying a *GBA1* mutation, compared with a similar‐sized control population.^1^ *GBA1* encodes for the lysosomal enzyme glucocerebrosidase (GCase), which catabolizes the sphingolipid glucosylceramide (GlcCer) to glucose and ceramide. Homozygous *GBA1* mutations result in the accumulation of GlcCer in lysosomes and cause the lysosomal storage disorder Gaucher disease (GD).^3^

The activity of GCase is decreased in PD brains with heterozygous *GBA1* mutations (PD+GBA), with the greatest decrease (58%) in the substantia nigra,^4^ the area of the brain with the greatest PD pathology. Notably, GCase was also significantly decreased by 33% in the substantia nigra of sporadic PD brains.^4^ GCase activity also has been reported to be decreased in the anterior cingulate cortex of sporadic PD brains.^5^

Defects in the autophagy–lysosomal pathway (ALP) are implicated in the accumulation/aggregation of α‐synuclein and mitochondrial dysfunction observed in PD.^6^, ^7^, ^8^ Cellular and animal models of GCase deficiency have shown impaired ALP, resulting in increased α‐synuclein levels and decreased mitochondrial function in neurons and brain.^9^, ^10^, ^11^, ^12^, ^13^ The mechanism by which loss of GCase activity affects the ALP is unclear. The accumulation of GlcCer or glucosylsphingosine (GlcSph; deacetylated GlcCer) could contribute to lysosomal dysfunction and toxicity, and so to the pathogenesis of PD in *GBA1* mutation carriers.^13^, ^14^ However, although PD+GBA brains have significant loss of GCase activity, the residual function of the enzyme may be sufficient to prevent substrate accumulation. Given the role of GCase in sphingolipid metabolism, we have performed lipidomic analysis of GlcCer, GlcSph, sphingomyelin, and gangliosides in the putamen and cerebellum of PD+GBA brains that we have previously reported to have decreased GCase activity.^4^

## Materials and Methods

### Postmortem Brain Material

Postmortem control brains, PD brains with known heterozygote *GBA1* mutations (PD+GBA), and sporadic PD brains were obtained from the Queen Square Brain Bank for Neurological Disorders after local ethical approval. *GBA1* mutations were identified by Sanger sequencing all 11 exons of the open reading frame. All donors gave written informed consent. All PD cases met the UK Brain Bank Clinical Criteria for Parkinson's disease. The pathological and biochemical analyses of these brains have been described.^2^, ^4^ Lipidomic analyses were performed on the putamen (n = 5‐7 per group) and cerebellum (n = 7‐14 per group). The genotypes of the PD+GBA putamen samples were one L444P/wt, one R463C/wt, one R193E/wt, and two N370S/wt. The genotypes of the PD+GBA cerebellum samples were five L444P/wt, one R463C/wt, one R131C/wt, three N370S/wt, one RecA456P/wt, one D409H/wt, one G193E/wt, and one Rec*NciI*/wt. The mean age (years ± standard error of the mean) for each cohort was: control, 65.4 ± 6.3; PD+GBA, 67.8 ± 3.0; sporadic PD, 69.6 ± 2.9. The postmortem delay (hours ± standard error of the mean) of each cohort: control, 55.1 ± 8.5; PD+GBA, 50.5 ± 6.6; sporadic PD, 37.8 ± 3.3.

### Total Cholesterol Measurement

Brain tissue was homogenized in isolation medium (250 mM sucrose, 10 mM Tris, pH 7.4, 1 mM ethylenediaminetetra‐aetic acid) and total cholesterol (free cholesterol and cholesteryl esters) measured using the Amplex Red Cholesterol Assay Kit (Life Technologies, Paisley, UK). Total cholesterol concentration (µg/mL) was normalized against the protein concentration of each sample (Pierce BCA Protein Assay Kit, Life Technologies, Paisley, UK).

### Lipidomic Analyses

Quantitative analysis of sphingolipids was performed by using liquid chromatography and tandem mass spectrometry. Briefly, tissues were homogenized in water to give a final concentration of approximately 100 mg/mL (w/v). The homogenate was extracted with 1 mL of a solution of acetonitrile:methanol:water (97:2:1, v/v/v) at room temperature. Extracts were injected onto an Atlantis HILIC silica column (Waters Corp, Milford, MA) for separation of GlcCer and GalCer, and these molecules were detected by using multiple reaction monitoring (MRM) mode tandem mass spectrometry with an AB Sciex API‐5000 mass spectrometer (AB Sciex, Framingham, MA). For other lipid analysis, extracts were injected onto an Acquity BEH C8 column (Waters Corp., Milford, MA), and MRM mode detection was performed using an AB Sciex API‐5000 mass spectrometer. For GlcSph analysis, homogenate was extracted with 1 mL of acetonitrile:methanol:water (48.5:50.5:1, v/v/v), and extracts were injected onto an Acquity BEH HILIC column to resolve GlcSph from psychosine, (Waters Corp., Milford, MA) and detected using MRM mode with an Agilent 6490 mass spectrometer. Except for phosphatidylcholine, all analytes were quantitated against standards obtained from Matreya, LLC (Pleasant Gap, PA).

## Results

Lipidomic analyses were performed on the putamen and the cerebellum. The substantia nigra was not measured because of insufficient material. In a previous biochemical study, the activity of GCase has been reported to be significantly decreased (*P* < 0.01 vs. control) by 48% in the putamen of PD+GBA brains and 47% in the cerebellum.^4^ In sporadic PD, GCase activity was decreased by 19% (nonsignificant) and 24% (*P* < 0.05 vs. control) in the putamen and cerebellum, respectively.^4^

**Figure 1:**
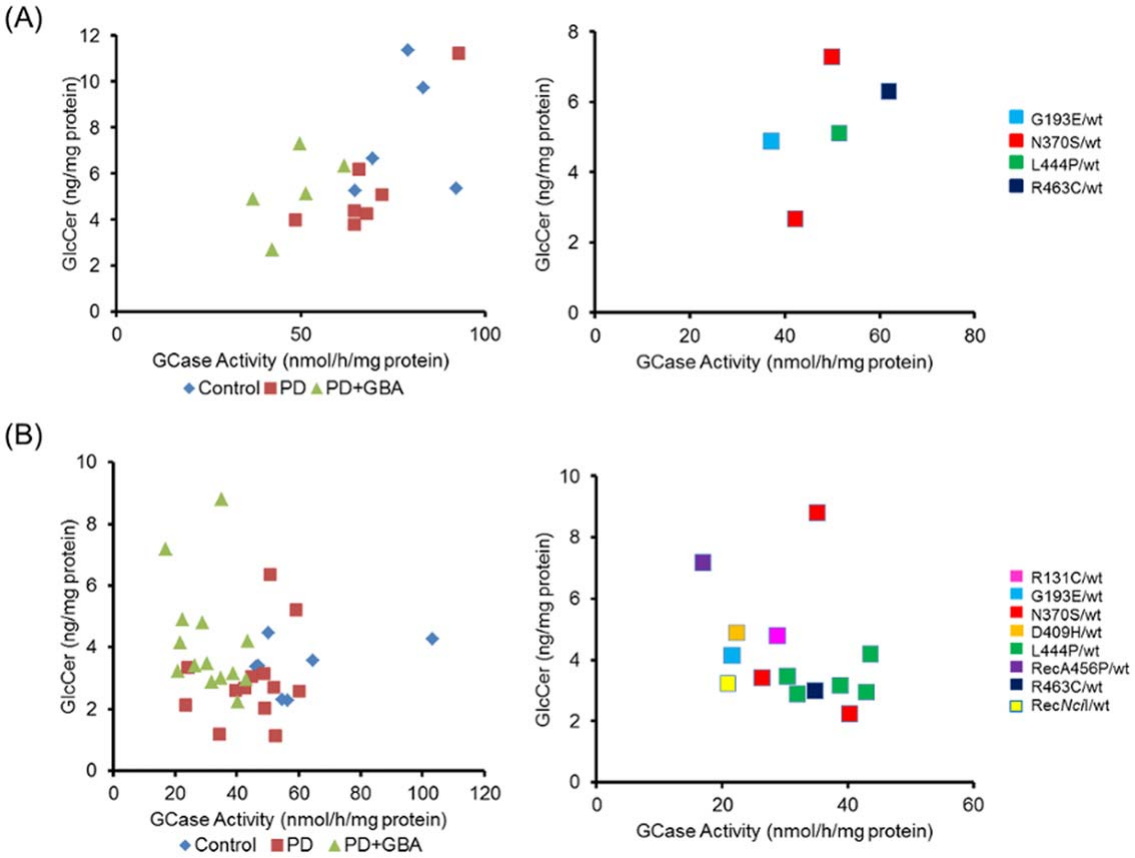
Plot of GCase activity against GlcCer levels for control, PD+GBA, and PD samples in putamen (**A**) and cerebellum (**B**). Relationship between GCase activity^4^ and GlcCer for each heterozygote *GBA* mutation is shown in the right panel. [Color figure can be viewed in the online issue, which is available at wileyonlinelibrary.com.]

Total GlcCer levels in the putamen and cerebellum were similar in control, PD+GBA, and sporadic PD brains when normalized against protein (Table 1). No changes were observed when total GlcCer levels were normalized to sphingomyelin in putamen or cerebellum (data not shown). Plotting total GlcCer levels against GCase activity for each individual did not show any correlation either (Fig. 1). Similar to previous studies, the predominant GlcCer species in human brain was C18:0.^15^, ^16^ The GlcCer C24:0 species was significantly increased in PD+GBA cerebellum by 212% (*P* < 0.05), when compared with control. No other changes in GlcCer species were observed in PD+GBA or sporadic PD brains.

**Table 1:** Sphingolipid and ganglioside levels unchanged in PD+GBA brains

Lipid	Putamen			Cerebellum		
	Control (n = 5)	PD+GBA (n = 5)	PD (n = 7)	Control (n = 7)	PD+GBA (n = 14)	PD (n = 13)
Total GlcCer	7.66 ± 1.23	5.25 ± 0.78	5.56 ± 0.99	3.38 ± 0.32	4.16 ± 0.48	2.94 ± 0.40
GlcCer C16:0	0.42 ± 0.10	0.31 ± 0.11	0.15 ± 0.03	0.29 ± 0.05	0.29 ± 0.04	0.19 ± 0.04
GlcCer C18:0	4.31 ± 0.88	2.73 ± 0.55	2.48 ± 0.11	2.08 ± 0.17	2.35 ± 0.33	1.57 ± 0.23
GlcCer C20:0	0.30 ± 0.08	0.19 ± 0.08	0.14 ± 0.03	0.18 ± 0.03	0.16 ± 0.03	0.15 ± 0.03
GlcCer C22:0	0.12 ± 0.03	0.16 ± 0.05	0.08 ± 0.05	0.01 ± 0.01	0.03 ± 0.01	0.00 ± 0.00
GlcCer C23:0	0.25 ± 0.03	0.16 ± 0.03	0.26 ± 0.08	0.05 ± 0.02	0.10 ± 0.02	0.07 ± 0.01
GlcCer C24:1	1.62 ± 0.21	1.21 ± 0.38	1.83 ± 0.65	0.61 ± 0.10	0.88 ± 0.15	0.74 ± 0.12
GlcCer C24:0	0.64 ± 0.06	0.49 ± 0.07	0.63 ± 0.22	0.17 ± 0.04	0.36 ± 0.04*	0.21 ± 0.04
GlcSph	0.14 ± 0.07	0.17 ± 0.08	0.35 ± 0.15	0.27 ± 0.06	0.20 ± 0.06	0.23 ± 0.06
LacCer	172 ± 53	203 ± 37	283 ± 81	219 ± 16	222 ± 22	213 ± 13
GM2 C18:0	386 ± 122	530 ± 53	498 ± 39	468 ± 30	468 ± 33	504 ± 45
GM3 C18:0	95 ± 41	135 ± 26	108 ± 24	90 ± 9	76 ± 10	103 ± 14
Sphingomyelin	26.4 ± 7.0	29.9 ± 2.0	31.6 ± 0.9	34.6 ± 1.2	36.7 ± 1.4	38.4 ± 2.6

Total glucosylceramide (GlcCer) and the different GlcCer species (C16:0, C18:0, C20:0, C22:0, C23:0, C24:0, C24:1, C24:0), glucosylspingosine (GlcSph) lactosylceramide (LacCer), GM2, GM3, and sphingomyelin were analyzed by LC‐MS/MS. Units are ng lipid/mg protein, except GM2/GM3, µg/mg protein; sphingomyelin, mg/mg protein. Data are mean ± SEM. *P < 0.05 vs. control as determined by one‐way analysis of variance followed by Tukey HSD test.

The levels of GlcSph have been shown to be increased in human GD brains and GD mouse models.^10^, ^14^, ^15^, ^17^ GlcSph was unaffected in the putamen and cerebellum of PD+GBA and sporadic PD brains, when compared with control.

No changes were seen in the amounts of lactosylceramide or sphingomyelin in either the putamen or cerebellum of PD+GBA or sporadic PD brains (Table 1). Although not significant, a trend was seen for increased ganglioside levels (GM2 and GM3) in the putamen of PD+GBA brains.

The levels of cholesterol were not significantly affected in the putamen (control, 375 ± 35 nmol/mg protein; PD+GBA, 328 ± 17; sporadic PD, 316 ± 22). The amount of phosphatidylcholine in the putamen was also unaffected (control, 20.93 ± 5.39 AU/mg of protein; PD+GBA, 23.49 ± 1.23 AU/mg of protein; sporadic PD, 25.36 ± 0.59 AU/mg of protein).

## Discussion

In this study, we found no evidence of accumulation of either GlcCer or GlcSph in the putamen or cerebellum of PD brains with heterozygote *GBA1* mutations. No changes were observed in sphingomyelin or cholesterol, although a trend was seen for increased GM2 and GM3 gangliosides in the putamen of PD+GBA brains. Possibly GlcCer accumulation is masked by shunting to these upstream gangliosides and other lipid species not analyzed in this study. We did not investigate ceramide, but both a strong trend for^5^ and a statistically significant^18^ decrease in total ceramide levels have been reported in sporadic PD anterior cingulate cortex,^5^ suggesting that changes in sphingolipid metabolism may occur in PD. Analysis of sphingolipids in regions of the limbic system with significant α‐synuclein pathology would be an interesting future study.

GCase activity was decreased by approximately 50% in the putamen and cerebellum of PD+GBA brains, with both regions showing a significant decrease in GCase protein expression.^4^ Heterozygous *GBA1* KO mice with a similar GCase deficiency do not show an increase in GlcCer or GlcSph in brain up to 6 months of age.^10^ Because the lipidomic analyses were performed on brain lysates, conceivably substrate accumulation in neurons is being masked by the more numerous glia. Subcellular fractionation also may indicate localized increases in GlcCer. Indeed, small increases in GlcCer have been reported in primary cultured cortical neurons with GCase knockdown (approximately 50% GCase activity loss)^9^ or dopaminergic neurons differentiated from inducible pluripotent stem cells harboring heterozygote *GBA1* mutations.^13^ However, in both cases, substrate accumulation was much less than the degree reported in type II/III GD brains.^15^

The mechanism by which GCase deficiency increases risk for developing PD is still unclear. In vitro experiments have suggested that GlcCer can stabilize soluble oligomeric forms of recombinant α‐synuclein at lysosomal pH.^9^ Although potentially relevant to GD patients who develop PD, this may not be the mechanism for PD+GBA patients lacking substrate accumulation in the brain. The GCase variant E326K has recently been reported to be the most common *GBA1* mutation in a British cohort of early‐onset PD cases (≤50 y of age).^19^ Although this mutation on its own decreases GCase activity, residual activity is still much greater than other GD mutations such as N370S and L444P.^20^, ^21^, ^22^, ^23^ Although still unproven, heterozygote E326K mutations causing GlcCer accumulation seems unlikely.

Because GCase is involved in sphingolipid metabolism, possibly changes in the composition of cellular membranes will contribute to PD. Impairment of the ALP has been suggested to contribute to the increased α‐synuclein observed in cellular and animal models of GCase deficiency.^9^, ^11^, ^13^ Membrane dynamics are critical for macroautophagy, ranging from biogenesis of the phagophore to the fusion of autophagosomes (APs) with lysosomes.^24^ Changes in the lipid content or cholesterol can impair the fusion of APs with lysosomes.^25^ Increased lysosomal cholesterol content or changes in the lipid composition also significantly inhibits chaperone‐mediated autophagy.^26^, ^27^ Therefore, although we or others have not found total changes in sphingolipid or cholesterol content in PD brains with *GBA1* mutations, sporadic PD brains, or GD brains,^5^, ^15^, ^28^ possibly the subcellular composition of organelles, lipid rafts, and other functional membrane domains are affected. Magnetic resonance spectroscopic imaging of PD patients with *GBA1* mutations has suggested that phospholipid metabolism is affected.^29^

## Author Roles

1. Research Project: A. Conception, B. Organization, C. Execution; 2. Statistical Analysis: A. Design, B. Execution, C. Review and Critique; 3. Manuscript Preparation: A. Writing the First Draft, B. Review and Critique.

M.E.G.: 1A, 1B, 1C, 2B, 3A, 3B

L.S.: 3B

B.H.W.: 1C

S.P.S.: 1A, 1B, 1C, 2B, 3A, 3B

A.H.V.S.: 1A, 1B, 3A, 3B

## Financial Disclosures

Drs. Gegg and Schapira report no disclosures.

Drs. Sweet, Wang, Shihabuddin, and Sardi are employees and stockholders of Genzyme, a Sanofi Company.
